# Epigenetics and life-long consequences of an adverse nutritional and diabetic intrauterine environment

**DOI:** 10.1530/REP-14-0334

**Published:** 2014-12

**Authors:** Nady El Hajj, Eberhard Schneider, Harald Lehnen, Thomas Haaf

**Affiliations:** 1 Institute of Human Genetics, Julius-Maximilians-Universität Würzburg, Biozentrum Am Hubland97074, Würzburg, Germany; 1 Department of Gynecology and Obstetrics, Städtische Kliniken, 41239 Mönchengladbach, Germany

## Abstract

The phenomenon that adverse environmental exposures in early life are associated with increased susceptibilities for many adult, particularly metabolic diseases, is now referred to as ‘developmental origins of health and disease (DOHAD)’ or ‘Barker’ hypothesis. Fetal overnutrition and undernutrition have similar long-lasting effects on the setting of the neuroendocrine control systems, energy homeostasis, and metabolism, leading to life-long increased morbidity. There are sensitive time windows during early development, where environmental cues can program persistent epigenetic modifications which are generally assumed to mediate these gene–environment interactions. Most of our current knowledge on fetal programing comes from animal models and epidemiological studies in humans, in particular the Dutch famine birth cohort. In industrialized countries, there is more concern about adverse long-term consequences of fetal overnutrition, i.e. by exposure to gestational diabetes mellitus and/or maternal obesity which affect 10–20% of pregnancies. Epigenetic changes due to maternal diabetes/obesity may predispose the offspring to develop metabolic disease later in life and, thus, transmit the adverse environmental exposure to the next generation. This vicious cycle could contribute significantly to the worldwide metabolic disease epidemics. In this review article, we focus on the epigenetics of an adverse intrauterine environment, in particular gestational diabetes, and its implications for the prevention of complex disease.

## Introduction

**Figure 1:**
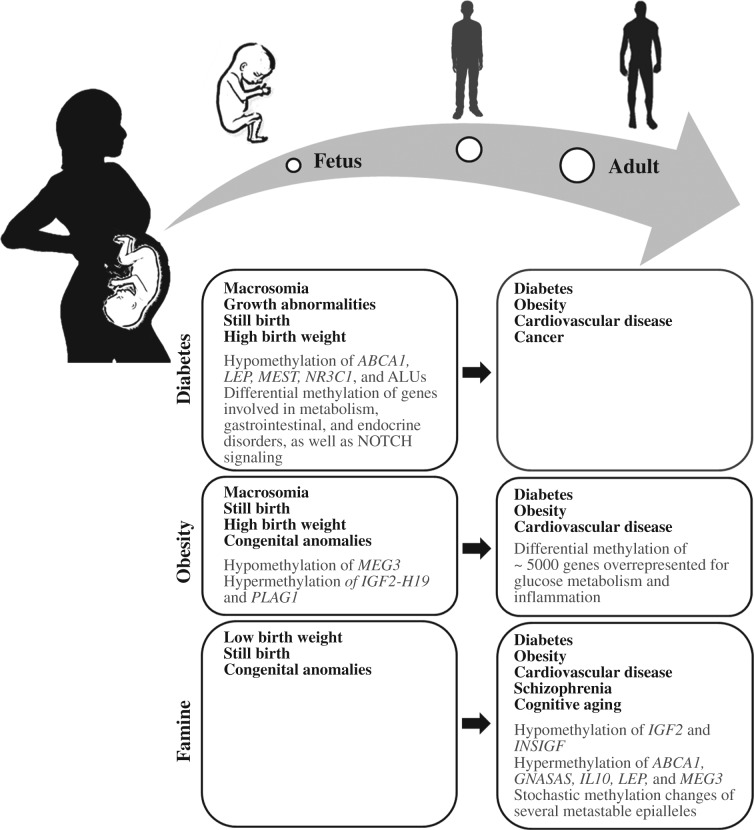
Medical problems and epigenetic changes (in placenta and blood) at birth and later in life, respectively, that have been associated with intrauterine exposure to diabetes mellitus, maternal obesity, and famine.

As early as 1880, puerperal diabetes has been associated with severe fetal and neonatal complications (Duncan 1882). In the 1950s, the term ‘gestational diabetes mellitus (GDM)’ was coined for a carbohydrate intolerance that develops during pregnancy and usually resolves after birth (Carrington *et al*. 1957). Over the course of pregnancy, increasing levels of hormones, such as lactogen, estrogen, and prolactin, are produced by the placenta to shift nutrients from the mother to the fetus and to prevent low blood glucose levels by resisting the action of insulin. Typically, insulin resistance arises during mid-gestation and progresses toward late-gestation. To counteract the diabetogenic effects of placental hormones, the mother's pancreatic islets try to increase insulin production (up to three times) during pregnancy. When the maternal β-cells cannot adapt to the increased food supply to the fetus and increased insulin demands during late pregnancy, the blood glucose levels will rise, stimulating insulin production of the fetal pancreas (Perkins *et al*. 2007). In GDM pregnancies, the balance between increased insulin resistance and maternal insulin production is disturbed, mostly due to insulin secretion defects of pancreatic β islets (Buchanan & Xiang 2005, Perkins *et al*. 2007). This results in maternal hyperglycemia, fetal hyperinsulinism, and consequently fetal overnutrition. Insulin gets glucose into cells for energy production and functions as a fetal growth factor, which may explain the increased risk for growth abnormalities, macrosomia, and stillbirth in GDM pregnancies (Fig. 1). The Hyperglycemia and Adverse Pregnancy Outcome (HAPO) Study on 25 000 pregnancies showed a strong correlation between maternal glucose levels and various perinatal complications (HAPO Study Cooperative Research Group *et al*. 2008, Lowe *et al*. 2012). Effective treatment of maternal diabetes by diet or insulin therapy can prevent adverse pregnancy outcome. The recommended diet (without fasting) consists of 45% carbohydrate, 30–35% fat, and 20% protein (American Diabetes Association 2003).

The GDM shares a common etiology with type 2 diabetes (T2D). The mothers with GDM often develop T2D later in life (Kim *et al*. 2002). The prevalence of GDM varies among populations and can range from 1 to >10%, depending on ethnicity and diagnosis criteria (American Diabetes Association 2003, Ben-Haroush *et al*. 2004). The well-known risk factors for GDM include pre-pregnancy obesity, advanced maternal age, and a family history of diabetes. Low physical activity and poor diet increase GDM susceptibility (Buchanan & Xiang 2005). Several sequence variants in genes that modulate pancreatic β cell function and insulin resistance were identified in GDM patients (Robitaille & Grant 2008).

It is now widely accepted that the intrauterine environment influences key developmental processes and has long-lasting effects on health and disease. Already 35 years ago, Freinkel (1980) proposed the concept of ‘fuel-mediated teratogenesis’, postulating that increased nutrients, in particular glucose levels and the resulting fetal hyperinsulinism in GDM pregnancies, have both immediate and long-lasting adverse consequences for the offspring. Since Barker *et al*. (1993) first reported an association between low birth-weight, which can be considered as a surrogate marker for an adverse intrauterine environment, and increased susceptibilities for metabolic and other complex diseases later in life, this phenomenon is also referred to as ‘Barker hypothesis’ or alternatively ‘developmental origins of health and disease (DOHAD)’ (Gillman 2005). According to the ‘thrifty phenotype’ concept (Hales & Barker 1992), intrauterine undernutrition and overnutrition, respectively, program adaptations of the fetal metabolism to cope with an adverse postnatal environment which lacks or is enriched with suitable nutrients.

Discordant siblings, who were born before and after the mother developed T2D, provide compelling evidence that it is the intrauterine diabetic environment which conveys an increased risk for metabolic disease in the offspring (Dabelea *et al*. 2000). Additional epidemiological and experimental studies (Wright *et al*. 2009, Moore 2010, Wu *et al*. 2012) support the concept that intrauterine hyperglycemia predisposes the offspring to obesity, metabolic and cardiovascular diseases, and a variety of cancers (Fig. 1). This vicious cycle may help to explain the epidemic increase of metabolic diseases in most developed countries.

Non-genetic intergenerational transmission of the metabolic phenotype has been extensively studied in GDM rodent models of intrauterine hyperglycemia. The offspring of rats with mild diabetes, induced by streptozotocin injection, are prone to develop diabetes and show fetal endocrine changes down to the third generation (Aerts & van Assche 1979). Embryo transfer experiments revealed that an euglycemic intrauterine environment cannot alleviate the strong genetic predisposition of Goto-Kakizaki (GK) embryos to develop diabetes, whereas a hyperglycemic environment in GK foster mothers augments the diabetes risk in Wistar embryos that have a low genetic susceptibility (Gill-Randall *et al*. 2004). Pancreatic islet transplantation in pregnant diabetic rats could improve glucose homeostasis in the offspring (Aerts & van Assche 1992). In a streptozotocin-induced GDM mouse model, intrauterine hyperglycemia was associated with impaired glucose tolerance and abnormal insulin levels in the F1 and F2 offspring, which may be related with abnormal methylation and expression of the *IGF2*-*H19* locus in pancreatic islets and impaired islet structure and function (Ding *et al*. 2012). It was speculated that dysregulation of *IGF2*-*H19* in sperm contributes to sex-specific transgenerational epigenetic inheritance.

## Metabolic phenotype in later life depends on intrauterine nutrition

### Phenotypic effects of an adverse nutritional environment in humans

A large number of studies have associated exposures to materno-fetal undernutrition (due to wartimes and wrong political decisions during the last century) with health outcome in later life. The famous ‘Dutch famine birth cohort’ consists of more than 2000 singletons who were born between November 1943 and February 1947 in Amsterdam and systematically followed up since 1996. Under the Nazi embargo of the Western Netherlands in 1944, pregnant women had only 400–800 kcal/day, roughly a quarter of the recommended intake. Individuals exposed to undernutrition during early gestation exhibited an increased risk for metabolic, cardiovascular and other complex diseases, schizophrenia, and accelerated cognitive aging (Roseboom *et al*. 2006, Schulz 2010; Fig. 1). Similarly, individuals who were born in China from 1958 to 1961 during the ‘Great Leap Forward’ famine displayed higher susceptibility for schizophrenia than comparable pre- and post-famine cohorts (Song *et al*. 2009). Children born from 1968 to 1970 during the Biafra famine in Nigeria showed a significantly increased risk for obesity and its confounding maladies (Hult *et al*. 2010).

Fetal overnutrition due to an obesogenic maternal environment also influences the metabolic phenotype later in life. Maternal obesity increases the risk of developing insulin resistance during pregnancy and intrauterine exposure to both maternal obesity and GDM has been linked to increased gestational weight, adiposity, and T2D in youth (Boney *et al*. 2005, Poston 2012; Fig. 1).

### Epigenetic effects

If early-life exposures confer a life-long increased risk for obesity, diabetes, and other complex diseases, they must cause persistent changes in the metabolism, neuroendocrine functions, etc. underlying these disease susceptibilities. One possible explanation is that intrauterine malnutrition interferes with organogenesis and, thus, has a long-lasting negative impact on organ morphology and physiology (Svensson *et al*. 1992). Another plausible mechanism is environmentally induced epigenetic modifications, leading to persistent changes in gene regulation and pathways (Gluckman *et al*. 2009, Lehnen *et al*. 2013). Epigenetic modifications, i.e. in form of DNA methylation and histone modifications, can cause changes in gene expression, which are transmitted to daughter cells during somatic cell division and, perhaps also from one generation to the next. It is well known that the epigenome(s) is highly plastic, particularly during early development (Reik *et al*. 2001) and susceptible to internal (i.e. during differentiation) and external environmental cues (Faulk & Dolinoy 2011, Feil & Fraga 2012).

A large number of studies on gene–environment interactions focused on 5-methylcytosine, the most prominent epigenetic modification of DNA itself. In non-coding regions of the genome, most CpG sites are methylated to prevent retrotransposition activity and genome instability (Yoder *et al*. 1997). CpG islands in promoter regions are usually demethylated, and promoter methylation during development or disease processes is associated with gene silencing (Jaenisch & Bird 2003, Weber *et al*. 2007). In contrast, CpGs in gene bodies are methylated, which is thought to play a role in exon definition and alternative splicing (Gelfman & Ast 2013). Approximately 100–200 of our more than 20 000 genes are expressed in a parent-specific manner. Most of these imprinted genes contain or lie near CpG islands that display differential methylation patterns between the maternal and paternal alleles (Kelsey 2007). Imprinted genes are essential for the regulation of fetal and placental growth, somatic differentiation, as well as neurological and behavioral functions after birth (Reik *et al*. 2003) and frequently used as a model to study the epigenetic effects of environmental factors during early development (Denomme & Mann 2012, El Hajj & Haaf 2013).

More than 60 years after early gestational exposure, the Dutch famine individuals showed subtle hypomethylation of the imprinted *IGF2*-*H19* locus, compared with their unexposed sibling (Heijmans *et al*. 2008). A follow-up study on the same cohort reported sex- and exposure timing-dependent changes in blood methylation of several imprinted and non-imprinted genes (Tobi *et al*. 2009; Fig. 1). A genome-wide screening of Gambian children conceived during the nutritionally poor rainy season revealed stochastic methylation changes in several putative metastable epialleles (Waterland *et al*. 2010), compared with children conceived in the dry season. The methylation status of metastable epialleles is established during early development, depending on stochastic and environmental factors, and influences the phenotype in later life (Rakyan *et al*. 2002). Genome-wide blood methylation and expression analyses revealed that maternal obesity/fetal overnutrition also has broad effects on the offsprings’ epigenome (Guénard *et al*. 2013).

### Animal studies

The agouti viable yellow (A^vy^) mouse is a particularly impressive model for the phenotypic consequences of epigenetic changes during early development. Insertion of an intracisternal A particle upstream of the A^vy^ allele renders the locus susceptible to stochastic and environmentally induced methylation after fertilization. Methylation-dependent silencing of the metastable A^vy^ epiallele suppresses the A^vy^ phenotype, which is characterized by yellow coat color as well as predisposition to obesity, insulin resistance, and cancer (Duhl *et al*. 1994, Morgan *et al*. 1999). When fed a western-style diet, the offspring of obese and diabetic A^vy^ mice are more susceptible to develop glucose intolerance, insulin resistance, and hepatic steatosis (Li *et al*. 2013*a*), suggesting epigenetic transmission of the metabolic phenotype to the next generation.

In streptozotocin-induced diabetic mouse models, maternal hyperglycemia interfered with formation of endoplasmic reticulum in maternal oocytes and the resulting embryos, and was associated with time-dependent methylation changes in several imprinted genes (Ge *et al*. 2013*a*, Zhang *et al*. 2013). The effects of an adverse intrauterine environment on oocytes from female offspring appeared to depend on the type of malnutrition. A high-fat maternal diet induced aberrant methylation changes across several metabolism-related genes in both maternal and fetal oocytes, whereas a hyperglycemic environment did not affect fetal germ-cell methylation patterns (Ge *et al*. 2013*b*, Ge *et al*. 2014). In addition, intrauterine exposure to either high-fat diet or maternal diabetes was shown to influence histone H3 and H4 acetylation of embryonal genes implicated in neural tube defects (Salbaum & Kappen 2012).

Similar to an intrauterine environment enriched with nutrients, early postnatal overfeeding can also influence the epigenetic and metabolic status of the offspring. In the small litter Wistar rat model, postnatal overnutrition caused rapid weight gain, early dyslipidemia, hyperinsulinemia, and dysregulated glucose homeostasis (Plagemann *et al*. 1992). The metabolic syndrome phenotype was associated with hypermethylation of the pro-opiomelanoctin (*Pomc*) and the insulin receptor (*Insr*) promoters in the hypothalamus (Plagemann *et al*. 2009, 2010). Small litter/overfed mice displayed similar pathophysiological changes as displayed by rats, along with a female-specific reduction in physical activity and energy expenditure. Genome-wide methylation analysis revealed persistent and sex-specific epigenetic changes across 900 loci in the hypothalamus associated with neural development (Li *et al*. 2013*b*).

Last but not least, the developmental origin of metabolic disease has also been studied in primates. Japanese macaques exposed to a high-fat maternal diet during pregnancy exhibited increased triglyceride levels and histological changes reminiscent of non-alcoholic fatty liver disease. These metabolic alterations were associated with hyperacetylation of several histone H3 variants which may be due to reduced fetal histone deacetylase 1 activity (Aagaard-Tillery *et al*. 2008).

## Fetal programing by GDM

In GDM pregnancies, the fetus is exposed to increased levels of glucose, free fatty acids, and amino acids, leading to increased fetal insulin production. Hyperglycemia and fetal hyperinsulinism are thought to program metabolic disease predispositions, which manifest later in life (Fig. 1). One plausible mechanism underlying fetal programing by GDM is that nutrient and hormonal levels in utero affect metabolic and neuroendocrine feedback loops, which by themselves are metastabile, but may be stabilized by epigenetic modifications that once established during development are further propagated throughout cell divisions.

### Candidate gene studies

Although genome-wide epigenetic studies have become feasible, the candidate gene approach is still valuable, because it avoids the multiple testing problem and, therefore, allows one to detect significant changes of small effect size. Bouchard *et al*. (2010) measured DNA methylation of leptin (*LEP*), a hormone that controls energy intake and expenditure, as well as body weight, in pregnant women with impaired glucose tolerance (Fig. 1). Maternal blood glucose levels correlated positively with *LEP* methylation in maternal placental tissue and negatively in fetal tissue. A follow-up study examined placental methylation of the adiponectin (*ADIPOQ*) gene promoter, an adipocyte-derived hormone with insulin-sensitizing effects (Bouchard *et al*. 2012). Lower *ADIPOQ* methylation in fetal placenta was associated with higher maternal glucose levels and lower methylation on the maternal side of the placenta with higher insulin resistance index. Houde *et al*. (2013) studied promoter methylation of ATP-binding cassette transporter A1 (*ABCA1*), a regulator of cholesterol efflux pathways and atherosclerosis. *ABCA1* methylation levels in maternal placenta correlated negatively with maternal HDL-cholesterol levels and positively with maternal glucose levels. On the fetal side, placental *ABCA1* methylation correlated negatively with cord blood triglyceride levels, whereas cord blood methylation correlated negatively with maternal glucose levels.

In our own study (El Hajj *et al*. 2013), we compared fetal cord blood as well as placenta methylation levels of seven imprinted genes involved in pre- and postnatal growth, four genes involved in energy metabolism, one anti-inflammatory gene, one tumor suppressor gene, one pluripotency gene, and two repetitive DNA families between GDM and control pregnancies. DNA methylation was quantified by bisulfite pyrosequencing that permits highly accurate measurements at single-base resolution. Of the 14 analyzed genes/repeats, the maternally imprinted *MEST* gene, the glucocorticoid receptor *NR3C1,* and ALU interspersed elements (Fig. 1) showed significant hypomethylation in both tissues of newborns of mothers with either dietetically treated or insulin-dependent GDM, compared with controls without exposure to GDM. The largest effect was observed for *MEST*, which showed a 4% lower methylation in cord blood and a 6% lower methylation in fetal placenta of GDM pregnancies. Recently, it has been reported that *MEST* hypomethylation in fetal cord blood is also significantly associated with paternal obesity, suggesting transmission of sperm epigenetic signatures for obesity into the next generation (Soubry *et al*. 2013). Consistent with its suspected role in the programing of an increased metabolic disease risk, *MEST* methylation was also significantly lower in the blood of obese adults than in normal-weight (sex- and age-matched) controls (El Hajj *et al*. 2013). In addition, *MEST *methylation was negatively correlated with BMI and waist circumference, supporting a role for this gene in the development of diet-induced obesity (Carless *et al*. 2013). In the mouse model, loss of *Mest* imprinting has been linked to increased body weight and organ size (Shi *et al*. 2004) and its overexpression promotes fat tissue expansion and adipocyte enlargement (Nikonova *et al*. 2008).

### Genome-wide analyses

One study used a luminometric methylation assay based on methylation-sensitive restriction enzymes to quantify global DNA methylation in pregnancies with different maternal medical risks (Nomura *et al*. 2014). The placenta but not the cord blood methylation levels were slightly lower in pregnancies with GDM and higher in pregnancies with maternal obesity. Although it is possible that GDM and obesity have a greater impact on placenta than on cord blood and oppositely program the epigenome, these results are preliminary.

To systematically compare cord blood methylation patterns between GDM and non-GDM pregnancies on a genome-wide scale, we used Infinium Human Methylation 450K bead chips, which quantify methylation at >485 000 CpGs distributed across 99% of all RefSeq genes including promoter, 5’UTR, gene body, and 3’UTR. Although we observed numerous between-group methylation differences in the order of one percentage point, following adjustment for multiple testing by the false discovery rate not a single CpG site remained significant. Since the sample size in current genome-wide epigenetic screens does not meet genetic (GWAS) study standards, it is not unexpected that small effects in individual genes do not reach genome-wide significance. Nevertheless, functional annotation enrichment analysis can help to elucidate the biological meaning of such data sets polluted with false positives and negatives.

Ingenuity Pathway Analysis identified ‘Cancer’, ‘Respiratory Disease’, ‘Metabolic Disease’, ‘Cardiovascular Disease’, and ‘Endocrine System Disorder’ as the top five diseases and disorders and ‘Carbohydrate Metabolism, Cardiac Dilation, Cardiovascular System Development and Function’ as top associated network functions. Using Panther Pathway Analysis, the top five pathways ‘Notch Signaling’, ‘Gonadotropin Releasing Hormone Receptor’, ‘PDGF Signaling’, ‘Heterotrimeric G-protein Signaling’, and ‘Integrin Signaling’ as well as the top five biological functions ‘Multicellular Organismal Process’, ‘Metabolic Process’, ‘Developmental Process’, ‘Cell Communication’, and ‘Cellular Process’ were significantly enriched (after Bonferroni's correction for multiple testing) with differentially methylated genes.

In a conceptually related study using the same methylation array, Ruchat *et al*. (2013) compared the cord blood and fetal placenta methylomes in GDM vs non-GDM pregnancies. The top loci in cord blood were enriched for the pathways ‘Gastrointestinal Disease’, ‘Metabolic Disease’, and ‘Endocrine System Disorder’. In placenta, the enriched pathways included ‘Cardiovascular Disease’, ‘Metabolic Disease’, and ‘Psychological Disorder’. When only considering genes with significant changes in both tissues, the enriched pathways ‘Immunological Disease’, ‘Metabolic Disease’, and ‘Endocrine System Disorder’ were identified. Several differentially methylated genes were correlated with birth weight, linking GDM exposure to macrosomia.

In a genome-wide methylated DNA immunoprecipitation-chip study, comparing blood methylation in offspring from diabetic (T2D) and non-diabetic Pima Indian mothers, pathway analysis of differentially methylated gene promoters identified ‘maturity onset diabetes of the young’, ‘T2D’, and ‘Notch signaling’ (Del Rosario *et al*. 2014).

Collectively, existing genome-wide analyses support the view that intrauterine diabetic environment epigenetically influences pathways involved in complex diseases. Consistent with a multifactorial disease model, epigenetic changes were reported in a large number of genes, however no changes with large effect size. Owing to small sample sizes and limited statistical power, so far no novel gene(s) has been identified with genome-wide significance in such studies. Concerted efforts (multi-center epigenome studies) are needed to recruit and analyze materials from larger study populations.

### Limitations

DNA methylation patterns are copied (by DNA methyltransferase 1) during DNA replication, implying that environmentally induced perturbations that occur during early development can be transmitted through cell divisions to subsequent cell generations. In this light, epigenetic modulations of gene regulation provide a plausible mechanistic link between intrauterine environment and life-long disease risk (Faulk & Dolinoy 2011, Feil & Fraga 2012, Lehnen *et al*. 2013). However, so far there is only circumstantial evidence that methylation changes that are programed in utero persist into adulthood and are causing metabolic changes. Most studies have only looked at narrow time windows either early or late in life. At present we cannot exclude the formal possibility that the associated epigenetic changes are only secondary effects of the pathophysiological processes predisposing to complex diseases.

Epigenetic variation is regulated in a tissue- and developmental stage-specific manner. Each cell type/tissue in our body is characterized by a specific combination of active and silenced genes, resulting from a complex interplay of stochastic, genetic, and environmental factors. In humans, it is hardly possible to study the target tissues of intrauterine programing, which in the case of GDM include fetal pancreatic islets, adipose tissue, skeletal muscle, liver, and the hypothalamic–pituitary–adrenal axis. Instead, easily accessible tissues such as cord blood and placenta are used to extrapolate the epigenetic effects of an adverse intrauterine environment underlying metabolic programing in the target tissues. Because of the enormous between-tissue differences in DNA methylation patterns, it is a major challenge to identify informative epigenetic signatures in blood or placenta epigenomes and then confirm that they reflect at least to some extent methylation variation in the target tissues. To account for the fact that neither fetal cord blood nor placenta is a crucial target for GDM, in our study (El Hajj *et al*. 2013) we only considered genes with significant between-group differences in the same direction in both tissues as likely candidates for metabolic programing.

Moreover, the studied tissues are usually composed of many different cell types. Sometimes, it is difficult to exclude that minor methylation changes which have been linked to a specific intrauterine condition are merely due to changes in cell composition of the analysed tissue. For example, whole blood of newborns of mothers with GDM may vary in the differential blood cell counts from normal controls (Yeruchimovich *et al*. 2000). The development of bioinformatic algorithms using DNA methylation signatures to infer changes in the cellular composition of whole blood samples between individuals (i.e. cases and controls) is a promising strategy to overcome this problem (Houseman *et al*. 2012).

## Outlook

The developmental origins or Barker hypothesis has been supported by a large number of epidemiological and animal studies during the past two decades. It has been proposed that intrauterine programing of the fetal metabolism has evolved to enhance maternal fitness (Wells 2007). On the other hand, the ‘parental conflict hypothesis’ (Moore & Haig 1991) postulates a battle over the allocation of maternal resources during pregnancy and also after birth, using imprinted genes as arms. In general, paternally expressed (maternally methylated) imprinted genes promote growth by extracting maximal maternal resources for a given pregnancy, whereas maternally expressed (paternally methylated) genes restrict growth and allocate resources equally among all offspring, which in polyandrous species may come from different fathers. In this context, it is interesting to speculate that paternally expressed genes, such as *MEST* (El Hajj *et al*. 2013), are susceptible to an intrauterine environment enriched with nutrients (overgrowth situation), whereas maternally expressed genes are more important for adaptation to a nutritionally poor environment (growth retardation). In this light, imprinted genes are an obvious target of metabolic programing and can serve as models for studying the long-term effects of an adverse intrauterine environment.

Because embryonal and fetal development exhibit considerable species differences, in particular between humans and rodents, animal models can be useful for proof-of-principle studies but the specific results cannot be directly extrapolated to the human situation. In the mouse model, the expression of several imprinted genes in the liver of offspring was influenced by maternal nutrition during gestation or lactation, but the methylation of the respective imprinting control regions remained largely unchanged (Ivanova *et al*. 2012).

### Is it beneficial to prevent epigenetic changes *in utero*?

Mothers who develop GDM are usually treated by dietetic measures and/or insulin therapy to prevent fetal hyperglycemia and hyperinsulinism. The methylation changes that are observed in the offspring at birth may result from both GDM and its treatment. In humans, it is difficult to dissect different components of epigenetic variation. Changes in maternal nutrition and pharmacological interventions during pregnancy may have significant effects on the fetal epigenome. During the nutritionally depressed rainy season in Gambia, supplementation of the maternal diet with vitamins and minerals around the time of conception had wide-spread and persisting (at least into early infancy) effects on the epigenome of the offspring (Cooper *et al*. 2012, Khulan *et al*. 2012).

Bariatric surgery can be an effective treatment for morbid obesity and diabetes (Edholm *et al*. 2013). Studies comparing siblings conceived before and after surgical weight loss of the mother showed that children who were not exposed to maternal obesity had a lower risk of developing obesity, improved metabolic status, lipid profiles, and insulin sensitivity (Kral *et al*. 2006, Smith *et al*. 2009). Genome-wide analyses of siblings born before and after maternal weight loss found methylation and expression differences in ∼5000 genes enriched in functional categories for glucose metabolism, diabetes signaling, inflammation, and autoimmune disease (Guénard *et al*. 2013).

In a mouse model of diabetic pregnancy, a high-protein diet was shown to modulate the expression of diet-responsive genes and reduce placental abnormalities (Kappen *et al*. 2012). Such diet-induced adaptations of the placenta have been implicated in fetal programing (Godfrey 2002) and may help to normalize aberrant methylation patterns and life-long disease risk. However, the materno-fetal interface of the placenta and its role for fetal programing remain to a large extent a black box.

### Is it possible to reverse epigenetic modifications after birth?

Eventually, early pharmacological, nutritional, and/or behavioral interventions can be applied to reverse epigenetic changes which have been programed by an adverse intrauterine environment. However, systematic experimental and clinical testing of such treatments in human newborns is difficult to justify. Although breast feeding is usually recommended, early neonatal ingestion of milk from diabetic mothers appears to increase the risk for obesity and impaired glucose tolerance in the offspring (Plagemann *et al*. 2002), whereas breast feeding after the first week of life and its duration have no influence on the childhood risk (Rodekamp *et al*. 2005). This argues in favor of the notion that the early neonatal period is a particularly critical time window for therapeutical interventions to break the vicious cycle of metabolic malprograming in utero. In the rat model, it was demonstrated that different postnatal inventions, i.e. neonatal leptin (Vickers *et al*. 2005, Gluckman *et al*. 2007), pre-weaning growth hormone (Gray *et al*. 2013), and juvenile folic acid treatment (Burdge *et al*. 2009) can prevent the long-term metabolic consequences of fetal undernutrition.

### Epigenetic biomarkers for metabolic disease risk?

There are only a few studies demonstrating that epigenetic signatures measured after birth or in childhood may serve as biomarkers for metabolic disease later in life. The methylation status of the retinoid X receptor, alpha (*RXRA*) and nitric oxide synthase 3 (*NOS3*) promoters in umbilical cord tissue of healthy newborns were associated with childhood adiposity at 9 years of age (Godfrey *et al*. 2011). Peroxisomal proliferator-activated receptor-gamma-co-activator-1alpha (*PPARGC1A*) promoter methylation in the blood of 5–7-year-old children was temporally stable and predicted adiposity up to 14 years (Clarke-Harris *et al*. 2014). These findings provide a proof-of-principle for the utility of methylation markers in the management of metabolic disease risk and emphasize the need for genome-wide longitudinal studies on well-defined birth cohorts.

If modulations of the fetal epigenome can permanently increase an individual's risk of chronic disorders in later stages of life, we should be much more concerned about ensuring an optimum environment from conception to birth. Following the developmental origins hypothesis, the most important time for the action to prevent metabolic disease epidemics is prenatally and early postnatally (Lehnen *et al*. 2013), when the epigenome is still highly plastic (Gluckman *et al*. 2009). Unfortunately, so far there is little knowledge on how to estimate the quality of the intrauterine environment in a given pregnancy and how to improve it. Research in this direction is urgently needed.
